# Iodine-based contrast media, multiple myeloma and monoclonal gammopathies: literature review and ESUR Contrast Media Safety Committee guidelines

**DOI:** 10.1007/s00330-017-5023-5

**Published:** 2017-08-30

**Authors:** Fulvio Stacul, Michele Bertolotto, Henrik S. Thomsen, Gabriele Pozzato, Donatella Ugolini, Marie-France Bellin, Georg Bongartz, Olivier Clement, Gertraud Heinz-Peer, Aart van der Molen, Peter Reimer, Judith A. W. Webb, on behalf of the ESUR Contrast Media Safety Committee

**Affiliations:** 1 S.C. Radiologia Ospedale Maggiore, Piazza Ospitale 1, IT-34129 Trieste, Italy; 2 Department of Radiology, University of Trieste, Strada di Fiume 447, IT-34149 Trieste, Italy; 3 Department of Diagnostic Radiology 54E2, Copenhagen University Hospital Herlev, Herlev Ringvej 75, DK-2730 Herlev, Denmark; 4 Department of Medical and Surgical Sciences, University of Trieste, Piazza Ospitale 1, IT-34129 Trieste, Italy; 5 Department of Internal Medicine, University of Genoa, Genoa, Italy; 6 Unit of Clinical Epidemiology, IRCCS, Azienda Ospedaliera Universitaria San Martino-IST-Istituto Nazionale per la Ricerca sul Cancro, Largo Rosanna Benzi 10, IT-16132 Genoa, Italy; 7 Service Central de Radiologie Hôpital Paul Brousse 14, av. P.-V.-Couturier, F-94807 Villejuif, France; 8 Department of Diagnostic Radiology, University Hospitals of Basel, Petersgaben 4, CH-4033 Basel, Switzerland; 9 Department of Radiology, Assistance Publique-Hôpitaux de Paris, Hôpital Européen Georges Pompidou, 20, rue Leblanc, Paris Cedex 15, F-71015 Paris, France; 10 Department of Radiology, Zentralinstitut für medizinische Radiologie, Diagnostik und Intervention, Landesklinikum St. Pölten, Propst Führer-Straße 4, St. Pölten, AT-3100 St. Pölten, Austria; 11 Department of Radiology, C2-S, Leiden University Medical Center, NL-2300 RC Leiden, The Netherlands; 12 Radiology, Klinikum Karlsruhe, Academic Teaching Hospital of the University of Freiburg, Molkestreet 90, D-76133 Karlsruhe, Germany; 13 Department of Radiology, St. Bartholomew’s Hospital, University of London, West Smithfield, London, EC1A 7BE UK

**Keywords:** Multiple myeloma, Monoclonal gammopathies, Contrast media, Acute kidney injury, Renal failure

## Abstract

**Objectives:**

Many radiologists and clinicians still consider multiple myeloma (MM) and monoclonal gammopathies (MG) a contraindication for using iodine-based contrast media. The ESUR Contrast Media Safety Committee performed a systematic review of the incidence of post-contrast acute kidney injury (PC-AKI) in these patients.

**Methods:**

A systematic search in Medline and Scopus databases was performed for renal function deterioration studies in patients with MM or MG following administration of iodine-based contrast media. Data collection and analysis were performed according to the PRISMA statement 2009. Eligibility criteria and methods of analysis were specified in advance. Cohort and case-control studies reporting changes in renal function were included.

**Results:**

Thirteen studies were selected that reported 824 iodine-based contrast medium administrations in 642 patients with MM or MG, in which 12 unconfounded cases of PC-AKI were found (1.6 %). The majority of patients had intravenous urography with high osmolality ionic contrast media after preparatory dehydration and purgation.

**Conclusions:**

MM and MG alone are not risk factors for PC-AKI. However, the risk of PC-AKI may become significant in dehydrated patients with impaired renal function. Hypercalcaemia may increase the risk of kidney damage, and should be corrected before contrast medium administration. Assessment for Bence-Jones proteinuria is not necessary.

***Key Points*:**

• *Monoclonal gammopathies including multiple myeloma are a large spectrum of disorders.*

• *In monoclonal gammopathy with normal renal function, PC-AKI risk is not increased.*

• *Renal function is often reduced in myeloma, increasing the risk of PC-AKI.*

• *Correction of hypercalcaemia is necessary in myeloma before iodine-based contrast medium administration.*

• *Bence-Jones proteinuria assessment in myeloma is unnecessary before iodine-based contrast medium administration.*

## Introduction

Since 1954, a number of case reports have linked the use of high osmolality ionic iodine- based contrast media to the development of acute renal failure in patients with multiple myeloma (MM) and monoclonal gammopathies (MG) who had undergone excretory urography and/or, less frequently, retrograde pyelography, cholecystography or angiography [1, 2].

Concern about the use of iodine-based contrast medium in myeloma patients has been challenged since the 1980s, when it was suggested that the deterioration in renal function was due to dehydration, pre-existing renal failure, diabetes mellitus, hypercalcaemia and the use of nephrotoxic drugs, rather than to the iodine-based contrast medium [3].

However, many radiologists and clinicians still consider MM and MG a contraindication to contrast medium use. They often require checks for Bence-Jones proteinuria before contrast-enhanced CT examinations, and deny clinically indicated investigations in myeloma patients. A survey among 2,000 practicing radiologists, members of the American College of Radiology (ACR), showed that 36 % of them never consider the use of iodine-based contrast media in myeloma patients [4], and the ACR still considered MM a possible risk factor for post-contrast acute kidney injury as recently as 2016 [5].

In view of these facts, the ESUR Contrast Media Safety Committee (CMSC) considered it necessary to undertake a systematic review of the available literature about the incidence of post-contrast acute kidney injury (PC-AKI) in patients with MM and MG, in order to produce evidence-based recommendations for the use of iodine-based contrast media in these patients. This study and the resulting guidelines were extensively discussed by the CMSC academic members and were also reviewed by the representatives of the pharmaceutical companies who are consultants to the Committee. A consensus report was agreed by the CMSC academic members at their meeting in February 2017.

### Clinical features of monoclonal gammopathies

MG include a large spectrum of disorders characterised by proliferation of neoplastic plasma cells that synthesise an abnormal amount of monoclonal immunoglobulins or immunoglobulin fragments. The clinical manifestations and course of this group of disorders are heterogeneous ranging from benign gammopathies of undetermined significance (MGUS), which are stable for years and do not need any treatment, to aggressive myeloma with bone fractures, renal failure and impaired haemopoiesis at presentation and a poor response to any treatment.

Until 2014 MG were classified as MGUS, smouldering myeloma (SMM), symptomatic MM, solitary plasmacytoma and immunoglobulin light chain amyloidosis [6]. MGUS and SMM are typically asymptomatic, and were differentiated by the level of the monoclonal protein and of clonal plasma cells in the bone marrow. The diagnosis of MM required demonstrable evidence of damage caused by the neoplastic plasma cells: hypercalcaemia, renal failure, anaemia and osteolytic bone lesions, commonly referred to as CRAB features. This definition ensured that patients with MGUS were not subjected to unnecessary chemotherapy, but in most patients with SMM, who have true malignancy, it is only a matter of time before end-organ damage occurs.

In recent years, reliable markers have been identified that can distinguish patients with SMM who are at imminent risk of end-organ damage. Also, improvements in myeloma treatment over the last 10 years suggest that therapeutic intervention at the SMM stage in these high-risk patients may improve quality of the life and overall survival. The main changes in the new classification [7] include an updated definition of MM that includes not only CRAB but also: (1) bone marrow infiltration by clonal plasma cells of 60 % or greater; (2) a serum free-light chains (FLCs) involved/non-involved ratio of 100 or greater; (3) more than one focal bone or bone marrow lesion at least 5 mm in size at MRI. A creatinine clearance less than 40 ml/min is used as the cut off for renal failure. On the basis of this new classification, patients showing one or more of these abnormalities should be considered to have MM and should undergo specific treatment. Although an increased proportion of MM patients undergo treatment, there is a larger population of SMM with high levels of immunoglobulins having haematological follow-up without any treatment. In SMM and MM patients the high monoclonal FLCs can overwhelm the capacity of the proximal tubule to reabsorb them so that they reach the distal tubule where they interact with specific proteins generating myeloma casts. These casts may block glomerular flow causing tubular atrophy and interstitial fibrosis [8]. Although the risk of renal failure increases with increasing concentrations of FLCs in the urine, this does not occur in all patients. In fact, not all monoclonal FLCs are equally nephrotoxic: the kidney injury depends partly on the type of FLCs and partly on environmental factors. At present, it is not possible to identify the potential nephrotoxicity of particular FLCs.

As well as the urine concentration of FLCs, there are many other factors that increase the risk of kidney damage in SMM and MM. These factors are in part secondary to MM, such as hypercalcaemia, hyperuricaemia, coexistent amyloidosis, hyperviscosity or abuse of non-steroidal anti-inflammatory drugs, and in part to comorbidity such as pre-existing nephropathy, diabetes mellitus, hypertension and cardiovascular disease. In a recent paper [9], the prevalence of renal impairment in a large group of SMM (1,135 cases) was 20 % at presentation. However, the renal function of a large proportion of these patients (54 %) improved after anti-myeloma induction therapy. This indicates that renal damage is reversible with treatment and that reducing the FLCs is the most important factor in protecting renal function. The same study shows that if patients never recover their renal function, they have a very short overall survival and early mortality. The goal of treatment at any stage of the disease is to achieve the maximal possible response rapidly, with minimal toxicity, and to improve the patient’s performance status.

## Methods

For this systematic review, data collection and analysis were performed according to the guidelines of the PRISMA statement 2009 [10]. Eligibility criteria and methods of analysis were specified in advance. Cohort studies and case-control studies reporting changes in renal function in patients with MM or MG following administration of iodine-based contrast media were included. Both non-controlled and controlled investigations, defined as studies in which patients with MM or MG were directly compared with patients without myeloma from the same community, were included. Studies in which a relationship between contrast medium administration and renal function changes could not be confirmed were excluded.

### Search strategy

A systematic search in Medline and Scopus databases was performed for studies of renal function deterioration in patients with MM or MG after administration of iodine-based contrast media. The search strategy was peer reviewed by an information specialist (DU). A combination of (MeSH) terms such as acute kidney injury, multiple myeloma, monoclonal gammopathies and synonyms was used. The complete Medline and Scopus search strategies are shown in Table 1. The PubMed function ‘Cited references’ and reference lists of all included articles were screened for additional relevant literature. A database of retrieved articles was made using endnote X7.7.1 (Thomson Reuters), and all duplicates were removed.

**Table 1:** Search strategies

**Database: PubMed (** **http://www.ncbi.nlm.nih.gov/pubmed** **)**
DATE: no limits/last searched November 20 2016
Total: 53
Strategy:
(“Acute kidney injury”[mesh] OR “kidney diseases”[mesh] OR “acute kidney injury”[tiab] OR “renal function”[tiab] OR “nephropathy”[tiab] OR aki[tiab] OR “renal failure”[tiab] OR ARF[tiab] OR CIN[tiab]) AND (“contrast media”[mesh] OR “contrast media”[tiab] OR “contrast medium”[tiab] OR “contrast induced”[tiab]) AND (multiple myeloma[mesh] OR “myeloma”[tiab] OR “monoclonal gammopathies”[tiab])
**Database: Scopus (** **http://www.scopus.com** **)**
DATE: no limits/last searched November 20 2016
Total: 116 (26 excluding PubMed duplicates)
Strategy:
TITLE-ABS-KEY (“Acute kidney injury” OR “kidney diseases” OR “renal function” OR “nephropathy” OR “aki” OR “renal failure” OR “ARF” OR “CIN”) AND TITLE-ABS-KEY (“contrast media” OR “contrast medium” OR “contrast induced”) AND TITLE-ABS-KEY (“myeloma” OR “monoclonal gammopathies”)

### Selection of studies

The first selection was made independently by two reviewers (FS and MB) with 34 and 21 years of experience in diagnostic imaging, respectively. Selection was based on the title and abstract without blinding to the authorship or journal. Then, references in full text that were deemed potentially relevant by either or both of the reviewers were retrieved, and evaluated according to their eligibility criteria. Non-English language articles were reviewed by native-language speakers to determine eligibility. No restrictions were imposed in relation to study design. If eligibility was doubtful, articles were discussed by the reviewers and were included or excluded based on consensus.

### Data extraction and data analysis

Information from each reference was extracted by two reviewers (FS and MB) working independently. The studies were categorised based on (1) type (systematic review, review article, case report, cohort or case-control studies); (2) number of contrast studies and of patients investigated (both myeloma patients and control patients from the same population, when present); (3) type of iodine-based contrast medium and route of administration; (4) possible causes for renal function deterioration other than iodine-based contrast medium administration; and (5) number of unconfounded associations between contrast administration and renal function deterioration. Unconfounded cases of contrast induced acute kidney injury (CI-AKI) were defined as situations in which post-contrast acute kidney injury (PC-AKI) could not be explained by comorbidity or causes other than contrast medium administration. The reviewers then graded the methodological quality of cohort and case-control studies by using the Newcastle-Ottawa scale for non-randomised studies [11]. Discrepancies were resolved by consensus.

Finally, based on the literature and the CMSC consensus, each guideline was graded using the Oxford Centre for Evidence Based Medicine (OCEBM) 2011 evidence classification: Grade A: established scientific evidence, Grade B: scientific presumption, Grade C: low level of evidence [12]. Recommendations based on CMSC consensus only were given Grade D because they were based on expert opinion.

## Results

**Fig. 1:**
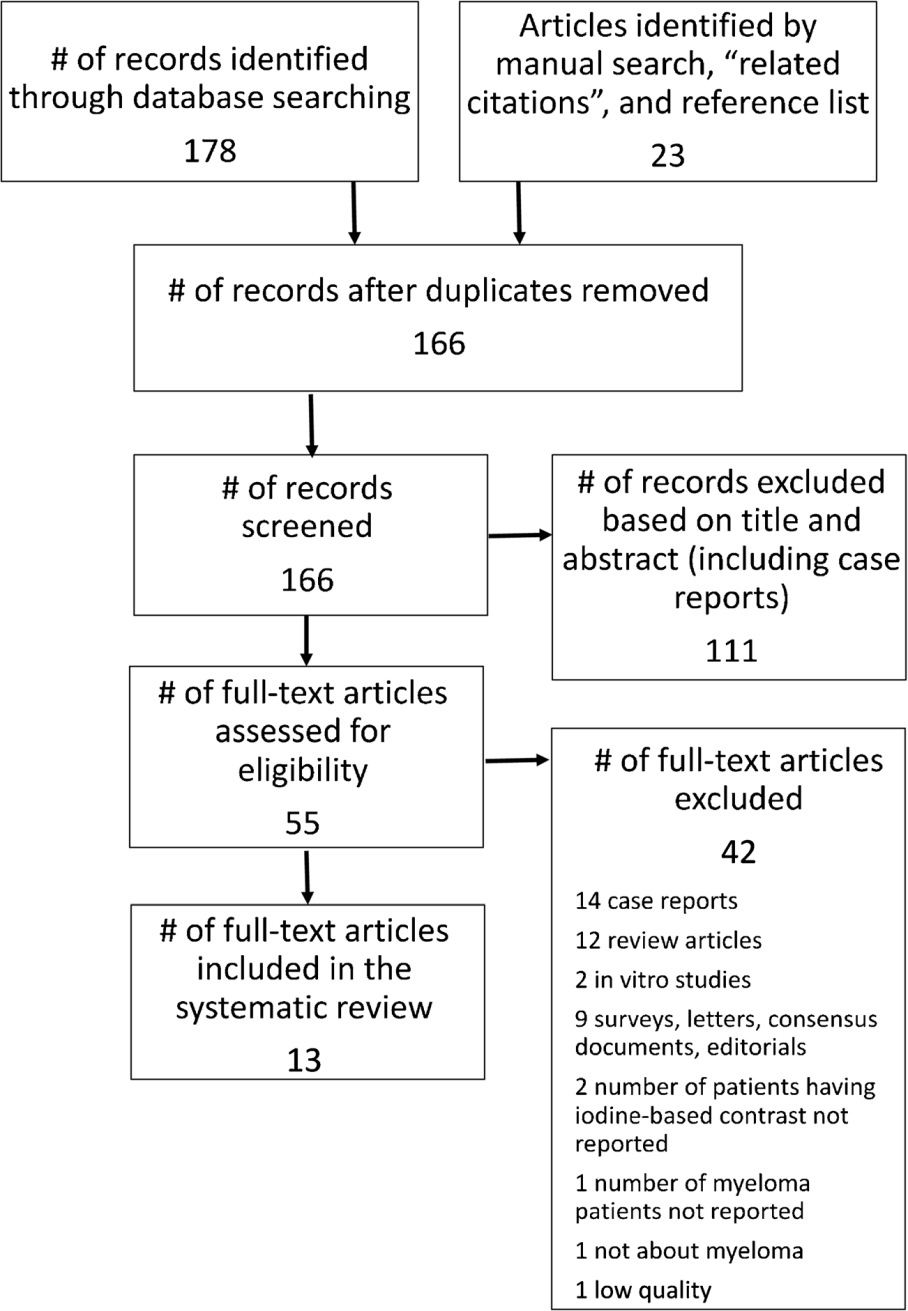
Search and selection procedures

The search and selection procedures used are shown in Fig. 1. Most studies were excluded during title and abstract review because they were not relevant to the clinical problem being studied. A total of 55 publications were considered potentially relevant and were retrieved in full text. Of these, 41 were excluded for a variety of reasons: 14 were case reports, 12 were review articles, two were in vitro studies in which no patients were investigated and nine were surveys, letters, consensus documents or editorials. In three studies the number of patients having iodine-based contrast medium or MM was not reported. The last paper was not about myeloma.

This left 14 observational studies that met the eligibility criteria used for this review [2, 13–25] (Table 2); 11 were retrospective and three were prospective [16, 21, 25]. Thirteen were cohort studies and one was a case-control study [25]. The Newcastle-Ottawa scores indicated quality levels ranging between 5 and 9 on a scale of grades 0–9 for cohort studies and 0–8 for case-control studies, except for one investigation [17] with a score of 1 (Table 3). This last study reported anecdotally on 18 intravenous urograms in 16 myeloma patients in whom no complications were observed and it was excluded from the systematic review. This left 13 studies with average Newcastle-Ottawa score grade 6.2 for evaluation. Of these, eight studies in which there was either no control group or the control group could not be evaluated [18] had lower scores of 5–6.

**Table 2:** Characteristics of included studies

Study, year	Study design	No. of exams/patients	Technique	Contrast medium type	Pathology	CIN (unconfounded)
Brown et al. 1964 [15]	Retrospective	39/46	U, 43 RP, 3	Iodopyracet,3 Sodium Acetrizoate, 1	Myeloma	4 *
Lasser et al. 1966 [17]	Retrospective	18/16	U	Unknown	Myeloma	0
Morgan et al. 1966 [18]	Retrospective Retrospective	19/18 105/105	U U	Unknown Unknown	Myeloma	0 # 0 #
Vix et al. 1966 [22]	Retrospective	52/40	U	Unknown	Myeloma	0
Svoboda et al. 1967 [24]	Retrospective	13/13 2/2	U C	Iodopyracet § Acetrizoate § Diatrizoate § Variety of contrast media §	Myeloma	0
Fateh-Moghadam 1969 [23]	Retrospective	55/69	U, 66 RP, 3	Diatrizoate (probably)	Monoclonal gammapathies	0¶
Myers et al. 1971 [19]	Retrospective	236/201	U, 218 U+RP, 18	Diatrizoate	Myeloma	0†
Defronzo et al. 1975 [2]	Retrospective	4/4	U	Unknown	Myeloma	1
Ansari et al. 1976 [13]	Retrospective	1**	U	Diatrizoate	Myeloma	0 ß
Baltzer et al. 1978 [14]	Retrospective	41/31	U	Diatrizoate, 28 ¥ Iothalamate meglumine, 14 ¥	Myeloma	0
Gassmann et al. 1983 [16]	Prospective	34/26	U	Iothalamate meglumine	Myeloma	0
Uchida et al. 1995 [21]	Prospective	13/10	U, 5 Unknown 8	Unknown	Myeloma	3
Pahade et al. 2011 [20]	Retrospective	80/46	CECT	Iodixanol, 10 Ioversol, 70	Myeloma	4
Preda et al. 2011 [25]	Prospective	130/30	CECT	Iodixanol	Monoclonal gammapathies	0

*All patients had severe proteinuria. Three, possibly all, had renal failure before urographic examination. Two developed urinary tract infection after retrograde pyelography

# Two independent patient series from two different hospitals

§ Iodine-based contrast administered to the single patients unknown

¶ Three confounded cases in patients with multiple comorbidities, dehydration and renal failure

† One confounded case in a patient with pre-existing renal failure who developed ARF

ß 25 patients with CIN. One patient with confounded CIN had myeloma. CIN is confounded in this patient because the patient had fluid deprivation the night before the examination, and septicaemia, and it is not clear whether this was already present at the time of urography

¥ Number of patients extrapolated from reported percentages

*CECT* contrast-enhanced CT, *U* urography, *RP* retrograde pyelography, *ARF* acute renal failure, *C* cholangiography

**Table 3:** Quality of studies

Study	Study design	Average Newcastle-Ottawa score
Brown et al. 1964 [15]	Retrospective cohort	5
Lasser et al. 1966 [17]	Retrospective cohort	1
Morgan et al. 1966 [18]	Retrospective cohort	6
Vix et al. 1966 [22]	Retrospective cohort	8
Svoboda et al. 1967 [24]	Retrospective cohort	5
Fateh-Moghadam 1969 [23]	Retrospective cohort	5
Myers et al. 1971 [19]	Retrospective cohort	7
Defronzo et al. 1975 [2]	Retrospective cohort	5
Ansari et al. 1976 [13]	Retrospective cohort	5
Baltzer et al. 1978 [14]	Retrospective cohort	9
Gassmann et al. 1983 [16]	Prospective cohort	5
Uchida et al. 1995 [21]	Prospective cohort	8
Pahade et al. 2011 [20]	Retrospective cohort	5
Preda et al. 2011 [25]	Prospective case-control	8

The 13 selected studies were markedly heterogeneous both in relation to type of investigation and contrast medium (Table 2). They report a total of 824 iodine-based contrast medium administrations in 642 patients with MM (639 administrations in 543 patients) or MG (185 administrations in 99 patients), in which 12 unconfounded cases of post contrast acute kidney injury were found (1.6 %). In 11 of the studies high osmolality ionic contrast media were used. Low osmolality non-ionic contrast media were used in two studies in which contrast-enhanced CT was performed 210 times in 76 patients with myeloma (n=46) or gammopathies (n=30) [20, 25]. PC-AKI was observed in 4/210 (1.9 %) cases [20]. These four patients had normal creatinine levels ranging from 0.9 to 1.3 mg/dl before contrast medium administration, and these increased slightly within 48 h after contrast injection (range: 1.2–1.6 mg/dl).

## Discussion

Contrast-enhanced studies with iodine-based contrast media are not routinely performed to stage MM or to investigate MG, but may be required to detect complications of the disease. However, many radiologists withhold iodine-based contrast media from all patients with MM or MG because they are considered at very high risk of acute kidney injury [4]. Until 2016, the ACR Manual on Contrast Media (version 10.2) stated that “paraproteinaemias, particularly MM, are known to predispose patients to irreversible renal failure after high osmolality contrast media administration due to tubular protein precipitation and aggregation; however, there is no data predicting risk with the use of low osmolality or iso-osmolality agents” [5]. This statement no longer appears in the 2017 ACR Manual (Version 10.3) [26]. As early as 1992 McCarthy and Becker [27] reviewed the literature on renal failure after high osmolality contrast media, and stated that major risk factors for acute renal failure in myeloma patients were not intravenous contrast media as once suspected, but rather hypercalcaemia, dehydration, infection and Bence-Jones proteinuria. They also noted that it was uncertain whether high osmolality contrast further increases these risks and that some of the older contrast media precipitate Bence-Jones protein in vitro.

Our review showed that the literature available is both limited and heterogeneous. There are a number of case reports. De Fronzo et al. [2] in 1975 collected 29 published case reports of acute renal failure in patients with MM who underwent intravenous urography (16 apparently with baseline normal renal function and 13 with chronic renal insufficiency). No randomised and very few controlled trials are available, and most studies are retrospective. Many studies were published before 1980. Most of the myeloma patients had intravenous urography after preparatory dehydration and purgation and ionic high osmolar contrast media that have been withdrawn from the market in most countries were used. Also, a variety of imaging techniques were used and there were no studies using intra-arterial contrast injection. Furthermore, different criteria for detecting post-contrast renal function deterioration were used. Only two studies considered the use of the non-ionic contrast media that are in wide use currently in patients with MM and MG. All these features limit the value of our systematic review.

The recent retrospective study by Pahade et al. [20] showed that the incidence of AKI following non-ionic contrast medium administration (ioversol or iodixanol) in patients with MM with a normal SCr is low and correlates with β_2_-microglobulin levels (which increase both with higher tumour burden and diminished renal function). Additionally, Preda et al. [25] carried out a prospective study showing that the use of a non-ionic dimer (iodixanol) is safe in patients with MG and an eGFR ≥60 ml/min/1.73 m^2^.

Although the evidence provided by the literature is rather poor, it appears that MM and MG alone cannot be considered a risk factor for AKI following intravenous iodine-based contrast medium administration. However, the risk may become significant when SMM and MM are associated with impaired renal function [8]. It therefore appears that, in cases of SMM or MM with normal renal function, it is not necessary to measure the urinary light chains before iodine-based contrast administration. In fact, if renal function is preserved, urinary light chains are unable to cause renal damage whatever their concentration. Therefore, in patients with normal renal function there is no need to check for Bence-Jones proteinuria, since this test cannot be considered as a surrogate biomarker of kidney function [8, 20]. When there is renal impairment, the usual rules for preventing PC-AKI should be considered, such as the possibility of an alternative imaging method not using iodine-based contrast media and volume expansion [28]. If contrast media are required for clinical reasons in patients with SMM or MM, measuring the serum calcium is much more important than checking the urinary FLCs. High concentrations of serum calcium are relatively common in any stage of SMM or MM, and are an important risk factor for AKI. Hypercalcaemia induces vasoconstriction and can inhibit antidiuretic hormone activity, so causing dehydration, which further increases the risk of myeloma casts, leading to worsening of the renal impairment [8]. Haematologists are fully aware of the risk of hypercalcaemia in SMM and MM, and serum calcium measurement is now one of the tests routinely performed in the follow-up of all stages of the disease. Also, hypercalcaemia is usually symptomatic causing malaise, constipation, anorexia, nausea, lethargy, confusion, coma and cardiovascular manifestations such as shortening of the QT interval and dysrhythmias. Therefore, it is very unlikely that an imaging method requiring iodine-based contrast medium administration will be requested without knowledge of the patient’s serum calcium. The treatment of hypercalcaemia is usually easy and includes aggressive re-hydration followed by diuresis with frusemide, which increases urinary calcium excretion, and intravenous bisphosphonate (pamidronate or zoledronic acid).

## Conclusion

Modern non-ionic iodine-based contrast media can safely be administered to patients with MM or MG who have normal renal function. However, comorbidity, such as impaired renal function and hypercalcaemia, which increases the risk of AKI, frequently coexists in these patients and close cooperation between radiologists and referring clinicians is needed for optimal management of these patients. Simple guidelines are proposed (Table 4).

**Table 4:** Multiple myeloma and monoclonal gammopathy patients: ESUR CMSC guidelines

	Level of evidence (*)
• Multiple myeloma and monoclonal gammopathy patients with normal renal function are not at increased risk of PC-AKI provided that they are well hydrated and that low- or iso-osmolar iodine-based contrast media are used	B
• Multiple myeloma patients often have reduced renal function, and such patients are at increased risk of PC-AKI	A
• Multiple myeloma patients often have hypercalcaemia which can increase the risk of kidney damage. Correction of hypercalcaemia before contrast medium administration should be discussed with the haematologist	D
• Assessment for Bence Jones proteinuria before contrast medium administration is not necessary	A

*Level of Evidence is graded using the Oxford Centre for Evidence Based Medicine (OCEBM) 2011 classification: Grade A: established scientific evidence, Grade B: scientific presumption, Grade C: low level of evidence. Recommendations based on CMSC consensus only were given Grade D (expert opinion)

*PC-AKI* post-contrast acute kidney injury

## Abbreviations

ACR: American College of Radiology
CI-AKI: Contrast-induced acute kidney injury
CIN: Contrast-induced nephropathy
CMSC: Contrast Media Safety Committee
CRAB: Calcium, Renal, Anaemia, Bone
ESUR: European Society of Urogenital Radiology
FLCs: Serum free light chains
MeSH: Medical Subject Headings
MG: Monoclonal gammopathies
MGUS: Gammopathies of undetermined significance
MM: Multiple myeloma
PC-AKI: Post-contrast acute kidney injury
PRISMA: Preferred Reporting Items for Systematic Reviews and Meta-Analyses
SMM: Smouldering myeloma
